# Design Methodology of Automotive Time-Sensitive Network System Based on OMNeT++ Simulation System

**DOI:** 10.3390/s22124580

**Published:** 2022-06-17

**Authors:** Feng Luo, Bowen Wang, Zhenyu Yang, Ping Zhang, Yifei Ma, Zihao Fang, Mingzhi Wu, Zhipeng Sun

**Affiliations:** 1 School of Automotive Studies, Tongji University, Shanghai 201804, China; luo_feng@tongji.edu.cn (F.L.); 1811023@tongji.edu.cn (Z.Y.); 2080103@tongji.edu.cn (P.Z.); 2033531@tongji.edu.cn (Y.M.); fangzihao@tongji.edu.cn (Z.F.); 2 Nanchang Automotive Institute of Intelligence and New Energy, Tongji University (NAIT), Nanchang 330052, China; wumingzhi@naiine.com (M.W.); sunzhipeng@naiine.com (Z.S.)

**Keywords:** time-sensitive network, automotive Ethernet, communications simulation, traffic scheduling, OMNeT++

## Abstract

Advances in automotive technology require networks to support a variety of communication requirements, such as reliability, real-time performance, low jitter, and strict delay limits. Time-Sensitive Network (TSN) is a keyframe transmission delay-guaranteed solution based on the IEEE 802 architecture of the automotive Ethernet. However, most of the existing studies on automotive TSN performance are based on a single mechanism, lacking a complete and systematic research tool. At the same time, the design method should be considered from a global perspective when designing an automotive TSN system, rather than only considering a single mechanism that TSN applies to. This paper discusses the correspondence between traffic types and automotive scenarios and proposes a methodology to target the delay constraint of traffic types as the design goal of automotive TSN networks. To study the performance of automotive TSN under different mechanisms such as time-aware shaper (TAS), credit-based shaper (CBS), cyclic queuing and forwarding (CQF), etc., this paper also develops a systematic automotive TSN simulation system based on OMNeT++. The simulation system plays a crucial role in the whole methodology, including all applicable TSN standards for the automotive field. Lastly, a complex automotive scenario based on zonal architecture provided by a major motor company in Shanghai is analyzed in the simulated system; verifying TSN can guarantee real-time performance and reliability of the in-vehicle network.

## 1. Introduction

In-vehicle network technology is one of the key technologies for Intelligent and Connected Vehicles (ICVs). With the further development of automotive intelligence, traditional bus technology can no longer meet the needs of the future, and related breakthrough technologies are constantly being invented [1]. Automotive Ethernet is such a new technology that while the Time Sensitive Network (TSN) plays an important role in guaranteeing real-time performance and low jitter for automotive Ethernet, at present, the research on automotive TSN is still in the initial stage.

As an enhanced standard for Audio Video Bridging (AVB) evolution, the TSN standard can be applied in any cyber-physical system with time-sensitive requirements, such as industrial automation, automotive, aerospace, and so on. Soheil Samii, a member of the TSN working group and the OPEN Alliance, and Helge Zinner, a member of the IEEE, described the TSN standard that could be used for an automotive Ethernet in the future [2]. In addition, some profiles of the TSN working group have also promoted the application of TSN technology in various fields, although these standards are still in the draft stage [3]. According to some existing studies, industrial automation and the automotive industry will take the lead in adopting TSN as their mainstream networking method or backbone network [4].

Lucia Lo Bello et al. [5] have claimed that Model-based Engineering (MBE) and Component-based Software Engineering (CBSE) play a critical role in the development of automotive embedded systems. With the addition of time-sensitive network constraints, the development process of automotive embedded systems will become more complicated. Therefore, adding effective real-time scheduling and schedulability analysis techniques [6,7,8] in the development process can ensure the time predictability of automotive embedded systems. The greatest benefit that automotive TSN can bring to the in-vehicle network is to ensure the real-time performance of high-priority traffic in the case of mixed traffic.

However, there are still two major problems in the research of TSN in the automotive field. First, in the case of complex in-vehicle traffic requirements, in which TSN sub-standards are applicable to the automotive field, how to use these standards or mechanisms has become a research hotspot. TSN is a combination of a set of standards. All TSN standards can be divided into four categories according to their functions, clock synchronization, reliability, delay determinism, and resource management. Different industrial fields choose the appropriate TSN standard due to different needs. In automotive Ethernet communication networks, it is not only necessary to transmit safety-related control traffic but also non-safety-related audio and video traffic or other traffic. Second, how to design the in-vehicle TSN network system from the perspective of car designers should be studied. The automotive Ethernet traffic types are closely related to the usage scenarios of Ethernet traffic. The same scenario can involve many different traffic types, and the same traffic type can also be used in different automotive scenarios. Thus, the relationship between traffic types and the scheduling algorithm of the TSN system needs to be studied. Moreover, the design goal of the in-vehicle TSN system is critical, and the network performance of the TSN is also related to automotive electronic and electrical architecture (E/E architecture) and software architecture.

The motivations of this work are as follows.

First, to unify and simplify the design process of the automotive TSN communication system, a design methodology of the automotive TSN system should be studied considering all the key points such as market requirements, design goals, automotive E/E architecture, programming design, simulation analysis, and integration testing. The design methodology should also include the correspondence between automotive traffic types and traffic scenarios. In addition, there should be a simulation system to easily simulate the real-time performance of the designed automotive TSN communication system. Lastly, the architecture and data sources of the simulation should be from the real automotive industry rather than from the artificially constructed, and the real-time performance of the real TSN system should also be analyzed.

Based on the above considerations, a design methodology of an automotive TSN system based on OMNeT++ is proposed in this paper. The contributions of this paper can be summarized as follows:

1. The correspondence between traffic types and automotive scenarios is discussed to meet different scheduling requirements.
2. The critical parameters and procedures in the TSN design process are pointed out, and the core design goals of the automotive TSN communication system are determined.
3. A methodology for designing and developing the automotive TSN communication system is proposed.
4. A complete systematic automotive TSN simulation system is designed to analyze the performance with single or mixed TSN scheduling mechanisms and algorithms.
5. The performance of a complex automotive scenario based on zonal architecture provided by a major motor company in Shanghai is studied through the complete systematic automotive TSN simulation system.

The rest of this paper is organized as follows: Section 2 introduces the related work of automotive TSN, especially the existing software simulation system. Section 3 points out the traffic characteristics and design goals of the automotive TSN networks, forming the design methodology. Section 4 introduces a complete automotive TSN simulation system based on the OMNeT++ simulation tool and analyzes the performance of a specific automotive TSN application scenario through the TSN simulation system. Section 5 summarizes this paper.

## 2. Related Works

TSN standards can be divided into two categories according to properties. One is the basic standard, which focuses on a type of function, and many standards with less functional content are attached as appendices or are directly incorporated into a basic standard; the other is a profile standard to facilitate the use of various industries and fields. The IEEE TSN working group provides profile standards in the fields of audio and video, mobile fronthaul networks, automotive, aerospace, and industrial automation. The release time, release status, and application fields of TSN series standards are shown in Table 1.

Existing research on TSN standards is studied individually based on the functions of the TSN standards. These studies can be divided into research on time synchronization, research on schedulability and scheduling algorithms, and research on TSN network security.

In terms of research on time synchronization, Andreas Kern et al. discussed the clock synchronization mechanism in automotive networks by measuring the effects of different automotive ambient temperatures on the clock synchronization accuracy [24]. Young Seo Lee et al. studied the IEEE 802.1AS synchronization mechanism combined with the synchronization mechanism of FlexRay to ensure synchronization performance in automotive heterogeneous networks [25]. Marina Gutiérrez et al. used OMNeT++ to simulate the synchronization accuracy of IEEE 802.1AS [26], while Maryam Pahlevan et al. simulated IEEE 802.1AS-Rev based on the OPNET platform [27].

In terms of research on schedulability and scheduling algorithms, the network calculus theory is used to calculate the maximum end-to-end delay of SR traffic class for Credit-based Shaper (CBS) defined in IEEE 802.1Qav [28,29,30,31]. Luxi Zhao et al. also used the network calculus to test the schedulability of the TSN system [32,33], aiming at the worst-case delay (WCD) of the critical traffic under different overlapping conditions of transmission window defined in IEEE 802.1Qbv and then proposed improvements [34]. Sivakumar Thangamuthu et al. analyzed the performance for in-vehicle networking applications by comparing time-aware shaper (TAS), burst-limiting shaper (BLS) and peristaltic shaper (PS) with OMNeT++ [35]. Luca Leonardi et al. simulated the performance of cyclic queuing and forwarding (CQF) in ADAS and infotainment systems by OMNeT++ [36]. Lucia Lo Bello et al. evaluate the end-to-end performance of EDSched in automotive scenarios under different data rates (i.e., 100 Mbps, 1 Gbps, 10 Gbps) [37]. Juho Lee et al. used OMNeT++ to verify the performance of the preemption defined in IEEE 802.1Qbu in an intelligent driving sensor network [38].

In terms of research on TSN network security, Doğanalp Ergenç et al. discussed more than 30 potential security issues and threats to the TSN standard [39]. Feng Luo et al. proposed an anomaly detection system based on per-stream filtering and policing (PSFP) defined in IEEE 802.1Qci [40].

In these studies, OMNeT++ has become the most common tool to evaluate the performance of the automotive TSN communication system. However, the above research on TSN mechanisms applying automotive scenarios is either a performance analysis under a single mechanism or a comparison between two mechanisms. Few research studies consider the performance analysis of applying mixed mechanisms and how to design automotive TSN networks at a holistic level.

To verify whether OMNeT++ can realize the above ideas, all the TSN mechanisms that OMNeT++ [41] can achieve are investigated. In addition, there is another simulation tool, OPNET [42]. Table 2 summarizes and compares the TSN features implemented by each simulation environment.

A well-accepted TSN simulation framework based on OMNeT++ is CoRE4INET [47], which is based on the INET library [48]. It provides an architecture for simulation modeling of the real-time Ethernet protocol TTEthernet [49] and TSN sub-standards. CoRE4INET was originally used to support the TTEthernet simulation and was later extended to support IEEE 802.1Q protocols such as AVB and TSN. The current version of CoRE4INET can emulate credit-based shaping, Stream Reservation Protocol (SRP) for network configuration, Time-aware Shaper, and Per-stream Filtering and Policing defined in the IEEE 802.1Qci standard. The source code of the framework is completely opensource. However, the PSFP mechanism implemented in CoRE4INET is not completely consistent with the protocol; the function of Internal Priority Value (IPV) is missing. Moreover, there are a few critical scheduling mechanisms not implemented in the CoRE4INET framework, such as the Ethernet frame preemption mechanism defined by IEEE 802.1Qbu, the Cyclic Queuing and Forwarding defined by IEEE 802.1Qch, the Asynchronous Traffic Shaping (ATS) defined by IEEE 802.1Qcr, and the redundancy mechanism defined by IEEE 802.1CB for Frame Replication and Elimination for Reliability (FRER).

Another popular TSN simulation framework is NeSTiNg [43], which is also based on the INET framework of OMNeT++. It was proposed by members of the TSN working group. Only the time-aware shaper and frame preemption mechanism are supported in the NeSTiNg framework. At the same time, the source code of the framework is completely opensource and can be obtained from the opensource website.

TSimNet [50] is also a TSN simulation framework based on OMNeT++. Only TAS, FSPF, and FERE are provided in the TSimNet framework. However, the source code of the simulation framework is only published on the intranet of the School of Electronic Information of Siegen University, and the source code of the framework cannot be directly obtained.

In terms of OPNET, H. Baniabdelghany et al. proposed a simulation framework based on the OPNET platform, which supports TAS, PSFP, and FRER [45]. In addition, M. Pahlevan et al. proposed a simulation framework based on the dynamic configuration mechanism of SRP based on the OPNET platform. However, the code for this simulation platform is not publicly available [46].

## 3. Methodology

During the design of the automotive TSN networks, there is a typical parameter: traffic priority. In simple terms, TSN switches support up to 8 queues equivalently to 8 priorities. However, the traffic of the in-vehicle network is diverse, and the traffic has various characteristics such as transmission mode, transmission cycle, end-to-end delay, tolerance to loss, and criticality, as shown in Table 3.

Therefore, in the process of automotive TSN communication, it is necessary to perform a different Quality of Service (QoS) for traffic with different characteristics. Different QoS means a different traffic priority assignment or a different shaping mechanism. The main purpose of applying automotive TSN networks is to ensure the real-time performance of traffic. If meeting the time constraint of the traffic is taken as the core goal of the design for automotive TSN networks, the traffic of different car scenarios can be mapped to different traffic classes, such that only the timing constraints need to be put forward for the traffic class, and the design goals can be formulated according to the requirements. As shown in Table 4, traffic in different scenarios can be mapped to 8 priorities. In the table, Priority Code Point (PCP) is defined in the IEEE 802.1, which represents the priority.

**Figure 1:**
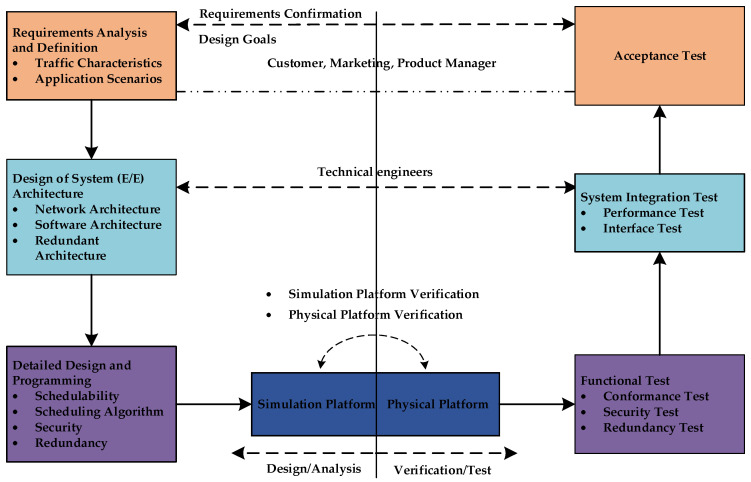
The methodology for the design of automotive TSN networks.

Considering the design of automotive E/E architecture, software architecture, the application scenarios and the traffic characteristics in this scenario, the methodology for the design of automotive TSN networks is formed, as shown in Figure 1, while the time constraint of traffic is taken as the core goal.

As shown in Figure 1, the design of automotive TSN networks mainly includes the following parts: requirements analysis and definition, design of the system or E/E architecture, detailed design and programming, simulation platform verification, physical platform verification, functional test, system integration test, and acceptance test.

The purpose of the requirement analysis and definition is to determine the application scenarios of the automotive TSN networks and all the traffic that need to be transmitted with characteristics requirements. This part of the work is generally carried out by customers, market personnel, and product manager, while all other stages require the participation of technical engineers. The design of E/E architecture consists of network architecture, software architecture, and redundant architecture. E/E architecture determines the structure of the entire TSN system. The network in the intelligent connected vehicles (ICVs) is a complex network coexisting with Ethernet, Controller Area Network (CAN), Local Interconnect Network (LIN), and other buses. In addition, the E/E architecture plays a decisive role in the performance of the network. The star topology and the ring topology have different advantages and applicable scenarios. Another aspect of the E/E architecture that needs to be determined is the redundant design of the network, including power redundancy and network redundancy. Generally, power redundancy is implemented by using additional power lines, while network redundancy is implemented by using redundant nodes or a ring network architecture. Finally, good software architecture can effectively reduce the delay of the signal at the software level inside the node.

The detailed design and programming are the core content of TSN, which mainly includes four parts: schedulability analysis, scheduling algorithm design, and security design and redundancy design. In the automotive TSN system, the schedulability analysis is used to verify the schedulable performance of the system under a given scheduling mechanism, and whether the application tasks meet the time requirements. The system can be verified in three different ways: formal mathematical model schedulability analysis, software simulation-based schedulability analysis, and physical-based schedulability analysis. The advantage of schedulability analysis can make designers choose a better shaping mechanism for automotive TSN networks. The design of the scheduling algorithm is related to the gate control list defined in IEEE 802.1Qbv. Designing a reasonable gate control state and gate control scheduling period is the key factor to realize the time determinism of the TSN. The security design serves the schedulability and the scheduling algorithm. The addition of security can ensure that the scheduling mechanism and the scheduling algorithm can work within a normal range without being affected by other attack traffic or abnormal traffic.

After everything is designed, the emulation platform can be used to simulate and verify the design of the automotive TSN networks. The emulation platform can be a software-based emulation platform or a hardware-based emulation platform. The hardware emulation platform can completely simulate the real automotive TSN system. Some standards that are still in the draft version or that will be released soon are not supported by switch chips; thus, all TSN standards can be implemented with the help of software emulation platform, and software emulation can greatly reduce the cost of the emulation platform.

The right-hand phases of the methodology are all testing phases, which include the functional test, system integration test, and acceptance test. The functional test is used to verify the conformance of the TSN standard, including the functional verification of the TSN scheduling mechanism, security redundancy, and time synchronization. The system integration test is used to verify the performance of the entire automotive TSN system and the conformance test of other protocols after integrating each TSN subsystem, including TCP/IP, Scalable Service-Oriented MiddlewarE over IP (SOMEIP), Diagnostic communication over Internet Protocol (DoIP) and other Ethernet-related protocols. Performance indicators such as maximum end-to-end delay, clock synchronization accuracy, clock synchronization time, system startup time, and link bandwidth utilization are tested during the performance test. Finally, regarding acceptance testing, all test results must meet the requirements.

## 4. Application Scenario

### 4.1. Simulation System

As mentioned above, only the software-based emulation platform can be shared by every designer throughout the design of the automotive TSN system. Other parts cannot be unified due to different car models, different requirements, different application scenarios, and different hardware devices. It is of great significance to establish a unified, systematic, and complete automotive TSN simulation platform

**Figure 2:**
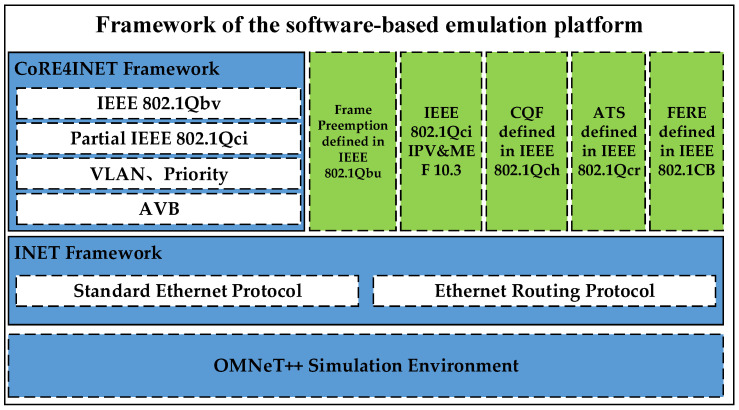
Framework of the software-based emulation platform.

The overall framework of the TSN software simulation platform designed in this paper is shown in Figure 2. The software simulation platform designed in this paper is based on the OMNeT++ simulator, the INET framework, and the CoRE4INET framework, adding frame preemption, IPV function, and token bucket algorithm in MEF10.3, cyclic queuing and forwarding, asynchronous traffic shaping as well as the redundant communication mechanism. In this case, the TSN simulation platform can simulate all TSN mechanisms applicable to the vehicle field and can analyze the schedulability of the system.

### 4.2. Use Case

**Figure 3:**
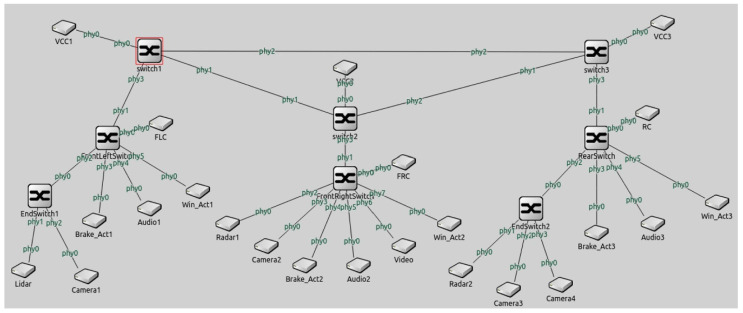
TSN topology in the simulation platform.

A TSN network model based on zonal architecture is designed in this paper. The zonal architecture and traffic information are provided by a major motor company in Shanghai. Traffic and architecture are slightly redesigned to simulate mechanisms-mixed time-aware shaper and redundancy. TSN topology in the simulation platform is shown in Figure 3.

E/E architecture of the automotive TSN system adopts a hybrid topology, including end systems, zonal controllers, domain controllers, backbone networks, and remote controllers. The backbone network adopts a ring topology, and the zonal controller and end systems adopt a star topology.

The communication among the end systems, zonal controllers, and backbone networks is established through 1000BASE-T1. The communication between the backbone networks and remote controllers is established through 1000BASE-TX. There is a total of three zones, which are distributed in the front left, front right, and rear of the car. To apply more complex scenarios, the design of functional domains (ADAS, Body, Chassis, and Infotainment) is assumed in each zonal network.

Sensors such as lidar, millimeter-wave radar, ultrasonic radar, and cameras are used in the ADAS domain. The brakes are controlled by motors in the Chassis domain. The infotainment domain is used to play video and 3D stereo surround music. In the body domain, motors are used to control equipment such as doors and windows.

The messages in the ADAS domain are designed as video and point cloud data from sensors such as lidar, millimeter-wave radar, ultrasonic radar, and cameras. The messages in the chassis domain are designed as brake control messages. The messages in the Infotainment domain are designed as video and 3D stereo surround audio messages. The messages in the body domain are designed as control messages to control the window.

According to the methodology for the design of automotive TSN networks, in the case of the above automotive application scenarios, traffic characteristics and E/E architecture, traffic class should be defined for the different traffic types. Therefore, the traffic priority can be defined according to Table 4, as shown in Table 5.

The complexity of this application is not yet the highest, because the number of traffic classes is less than eight, and messages with traffic class 1 and 2 can completely increase their priority. We reset the traffic priority of audio and video to 4, and the priority of window control to 3. The purpose of the simulation in this paper is not to target the most complex situation, but to carry out a detailed design according to the design methodology of the automotive TSN network and to analyze the performance of mechanisms-mixed time-aware shaper and redundancy. The whole traffic characteristics in this TSN network are shown in Table 6.

The total bandwidth without redundancy is 408.9 Mbps. Considering the shunting effect of three VCCs, the maximum aggregate bandwidth on the backbone network will also be lower than 408.9 Mbps. If redundant packets are added, the maximum bandwidth is 548.2 Mbps.

The next step is detailed design and programming. The performance of scheduling and redundancy is analyzed in this case. Because the number of traffic classes is less than eight, the -TAS defined in IEEE 802.1Qbv fully meets the requirements. We compared the end-to-end delay of each traffic after adding the scheduling mechanism and redundancy mechanism, including the average, minimum, and maximum conditions, as shown in Table 7.

As mentioned in the design methodology of the automotive TSN system, timing constraints are the core goal of the TSN design; thus, the main concerns of the performance analysis are the parameters related to time characteristics, such as the maximum end-to-end delay and the jitter. If the maximum end-to-end delay of traffic meets the time delay requirement, and the jitter is almost 0, then the TSN design is successful. The requirements of the time delay from the motor company are shown in Table 8.

It can be seen that the maximum end-to-end delay of the traffic Lidar is 61.49 μs/3 hops in the situation without applying scheduling and redundancy. The latency cannot meet the timing constraints defined in Table 8. Although the time delay of other traffic can meet the requirements, the jitters are still at a high level, especially the traffic Brake_Act3.

**Figure 4:**
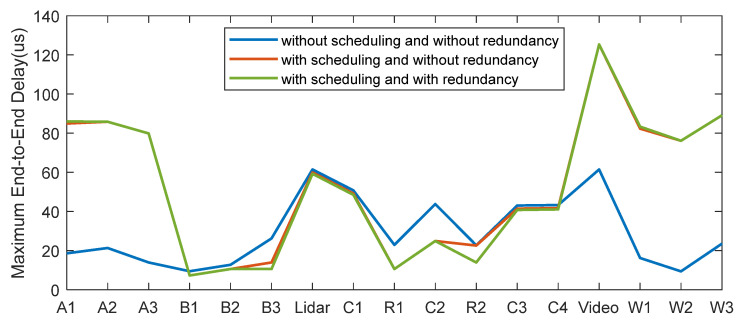
Maximum end-to-end delay of each traffic with different situations.

**Figure 5:**
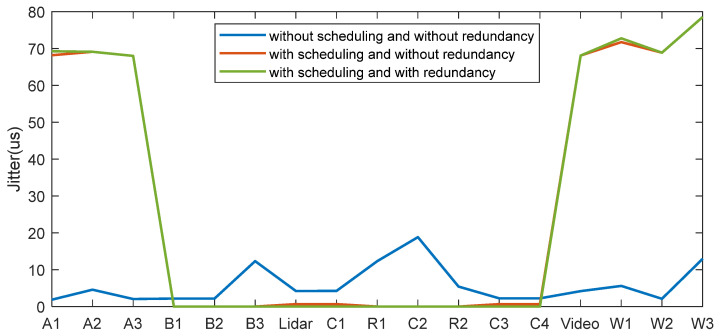
Jitter of each traffic with different situations.

As shown in Figure 4 and Figure 5. In the situation with applying the time-aware shaper, the jitter and the maximum end-to-end delay of higher-priority traffic (6, 7) are greatly reduced, increasing the time certainty. The latency of the traffic Lidar is 59.95 μs, meeting the timing constraints. However, it will slightly increase the jitter and the maximum end-to-end delay of lower-priority traffic by about 70 μs. It also increases the average delay of lower-priority traffic, which is acceptable. As a result, the real-time performance improvement of the high-priority traffic brought by TAS sacrifices the real-time performance of low-priority traffic.

The addition of the redundancy function has little effect on the end-to-end delay and jitter of each traffic under the bandwidth limitations. Redundancy can increase reliability by sacrificing a certain bandwidth. It can also be seen from the following analysis that the redundant packets will pass through the two paths. The packets that arrive first will be received, and the packets that arrive later will be discarded.

**Figure 6:**
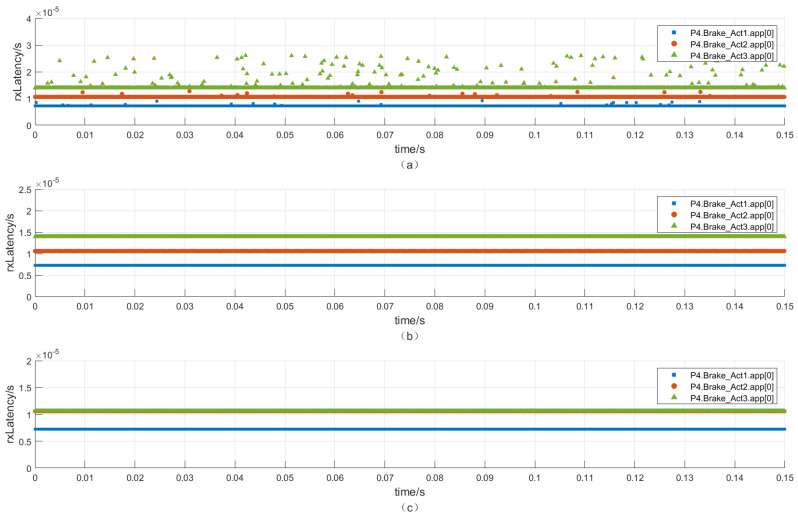
End-to-end delay of brake control traffic (**a**) without scheduling and without redundancy; (**b**) with scheduling and without redundancy; (**c**) with scheduling and with redundancy.

As shown in Figure 6, the three brake control traffics of the highest priority are concerned. It can be seen that when the redundancy function is not used, the minimum end-to-end delays of the three traffics are concentrated at the levels of 7, 10.6, and 14 μs. Because the number of hops traversed by the three traffics is different, when the redundancy function is used, Brake_Act3 has one path that goes through 3 hops and the other path that goes through 4 hops; thus, the traffic that passes through 3 hops will be accepted, and the traffic that passes through 4 hops will be eliminated. Therefore, the delays are concentrated at two levels of 7 and 10.6 μs.

When the scheduling mechanism is applied, the jitter of the brake control traffic will be reduced from the maximum jitter of 12.3 to 0 μs. The maximum jitter of 12.3 μs is because Brake_Act3 is affected by the video traffic when it is not scheduled.

As a result, the design of a TSN system based on the zonal architecture was successful. All the end-to-end delays and jitters of the traffic in this TSN system applying time-aware shaper and FRER can meet the timing constraints defined by the motor company in Shanghai.

## 5. Conclusions

In this paper, how to design a perfect or optimal automotive TSN system has been discussed, and the automotive TSN design methodology is proposed from a global and holistic perspective. Different car manufacturers have different development models, and each model may also use different in-vehicle network systems. However, the design of any automotive TSN system can follow the methodology presented in this paper regardless of the differences existing in different companies or researchers. In the methodology, the correspondence between traffic types and traffic priorities is critical. The priority of the traffic determines the scheduling algorithm and different QoS. The core design goal of the automotive TSN system is meeting the timing constraints of each traffic.

To verify the success of the design of the TSN system, the end-to-end delay and jitter for each traffic should be analyzed. Therefore, a complete and systematic automotive TSN simulation platform is designed in this paper to study the performance of further scheduling algorithms and mechanisms, including single and mixed mechanisms. All the scheduling mechanisms suitable for the automotive field can be analyzed in the simulation platform.

Finally, the performance of a TSN network for autonomous driving based on zonal E/E architecture provided by a major motor company in Shanghai was analyzed, containing various traffics from different domains (ADAS, Body, Chassis, and Infotainment). After applying the time-aware shaper and redundancy mechanism defined in TSN, the end-to-end delays of high-priority traffic can meet the timing constraints defined by the motor company, and the jitter of the control traffic and ADAS traffic was reduced to 0 μs.

In the future, we plan to analyze the performance of the asynchronous traffic shaper defined in IEEE 802.1Qcr and study which in-vehicle scenarios the mechanism is suitable for and how it works with other TSN scheduling mechanisms through the simulation platform that this paper presented. We also plan to optimize the TSN scheduling algorithm with the help of intelligent algorithms such as machine learning.

## Figures and Tables

**Table 1:** Development status of TSN standards.

Standard	Name	Status	Application Field			
			AV ^1^	AI ^2^	IA ^3^	MF ^4^
IEEE 802.1Qav-2009 [9]	Forwarding and Queuing Enhancements for Time-Sensitive Streams	Published	√	√	√	
IEEE 802.1Qca-2015 [10]	Path Control and Reservation	Published			√	
IEEE 802.1Qbv-2015 [11]	Enhancements for Scheduled Traffic	Published		√	√	
IEEE 802.1Qbu-2016 [12]	Frame Preemption	Published	√	√	√	√
IEEE 802.1Qch-2017 [13]	Cyclic Queuing and Forwarding	Published	√	√	√	
IEEE 802.1Qci-2017 [14]	Per-Stream Filtering and Policing	Published	√	√	√	
IEEE 802.1CB-2017 [15]	Frame Replication and Elimination for Reliability	Published	√	√	√	
IEEE 802.1Q-2018 [16]	Bridges and Bridged Networks	Published	√	√	√	
IEEE 802.1CM-2018 [17]	Time-Sensitive Networking for Fronthaul	Published				√
IEEE 802.1Qcc-2018 [18]	Stream Reservation Protocol (SRP) Enhancements and Performance Improvements	Published	√		√	
IEEE 802.1Qcp-2018 [19]	YANG Data Model	Published	√		√	
IEEE 802.1AS-2020 [20]	Timing and Synchronization for Time-Sensitive Applications	Published	√	√	√	
IEEE 802.1Qcr-2020 [21]	Asynchronous Traffic Shaping	Published		√	√	
IEEE 802.1CS-2020 [22]	Link-local Registration Protocol	Published	√		√	
IEEE 802.1DG [23]	Time-Sensitive Networking Profile for Automotive In-Vehicle Ethernet Communications	Drafted		√		

^1^ AV: audio video. ^2^ AI: automotive industry. ^3^ IA: industry automation. ^4^ MF: mobile fronthaul.

**Table 2:** TSN features implemented by each simulation environment.

TSN Features	OMNeT++			OPNET	
	NeSTiNg [43]	CoRE4INET [44]	TSimNet	H. Baniabdelghany et al. [45]	M. Pahlevan et al. [46]
Credit-based Shaper		√			
Stream Reservation Protocol		√			√
Time-aware Shaper	√	√		√	
Frame Preemption	√		√		
Per-Stream Filtering and Policing		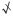 (Partial)	√	√	
Cyclic Queuing and Forwarding					
Asynchronous Traffic Shaping					
Frame Replication and Elimination for Reliability			√	√	
Source Code Availability	Available	Available	Not Available	Not Available	Not Available

**Table 3:** Traffic characteristics of the in-vehicle network.

Characteristics	Description
Transmission Pattern	In-vehicle traffic can be sent according to events or cycles
Transmission Period	The transmission period represents the data transmission interval planned by the application layer
End-to-end Delay	Indicates the time taken by the traffic from the sender to the receiver, and the maximum end-to-end delay is the maximum value of all end-to-end delays of the traffic
Tolerance to Loss	Tolerance to loss indicates the application’s tolerance for continuous packet loss in the network transmission of the traffic
Criticality	The criticality is expressed as the degree of impact that may be caused by not guaranteeing the real-time performance of the traffic, which can be divided into three categories: high, medium, and low.

**Table 4:** Correspondence between traffic types and automotive scenarios.

Traffic Class	PCP	Priority	Automotive Scenarios
TC8	7	Highest	Safety-related control signals, such as engine signals, brake signals, turn signals, Advanced Driving Assistance System (ADAS) control signals, etc.
TC7	6		Safety-related media signals, such as environmental perception sensor signals: millimeter-wave radar, lidar, ultrasonic radar, cameras, ADAS fusion data, real-time map download, positioning signals, etc.
TC6	5		Reserved
TC5	4		Network management signals, such as Precision Time Protocol (PTP) synchronization messages, network redundancy signals, network diagnostic signals, etc.
TC4	3		Vehicle to Everything (V2X) related events, warnings, alarm signals, dynamic network configuration signals, etc.
TC3	2		Non-safety-related control signals, such as lighting control, air conditioning control, door and window control, infotainment system control, etc., and vehicle status sensor signals: fuel battery consumption, water temperature, tire pressure signal, etc.
TC2	1		Non-safety-related media signals, such as audio and video signals of audio-visual entertainment systems, low-speed camera signals: reversing cameras, 360-degree surround-view cameras, head-up display signals (HUD), etc.
TC1	0	Lowest	Firmware Over the air technology (OTA) and software OTA, including offline map download, etc., cloud logging, uploading, diagnostic and configuration signals, and other Internet data access

**Table 5:** Design table of traffic priority and redundancy.

Domain	Traffic type	Traffic Class	Zone	Transmission Path
ADAS	Safety-related media signals	6	Front left	Lidar/camera -> Zonal Controller1 -> Central Controller1 -> Remote Controller1
			Front right	Radar/camera -> Zonal Controller2 -> Central Controller2 * -> Central Controller1 -> Remote Controller1
			Rear	Radar/camera -> Zonal Controller3 -> Central Controller3 * -> Central Controller1 -> Remote Controller1
Chassis	Safety-related control signals: brake signals	7	Front left	Central Controller1 -> Zonal Controller1 -> Brake Motor1
			Front right	Central Controller1 * -> Central Controller2 -> Zonal Controller2 -> Brake Motor2
			Rear	Central Controller1 * -> Central Controller3 -> Zonal Controller3 -> Brake Motor3
Infotainment	video	1	Front right	Video->Zonal Controller2 -> Central Controller2 -> Central Controller3 -> Remote Controller3
	3D stereo surround music	1	Front left	Remote Controller3 -> Central Controller3 -> Central Controller1 -> Zonal Controller1 -> Audio1
			Front right	Remote Controller3 -> Central Controller3 -> Central Controller2 -> Zonal Controller2 -> Audio2
			Rear	Remote Controller3->Central Controller3->Zonal Controller3->Audio3
Body	Non-safety-related control signals: door and window signals	2	Front left	Central Controller2 -> Central Controller1 -> Zonal Controller1 -> Window1
			Front right	Central Controller2 -> Zonal Controller2 -> Window2
			Rear	Central Controller2 -> Central Controller3 -> Zonal Controller3 -> Window3

* Applying redundant operation.

**Table 6:** Whole traffic characteristics in this TSN network.

Traffic Type	Source	Destination	Priority	VLANID	Size (Bytes)	Start Time	Transmission Interval (μs)	Redundancy	Bandwidth (Mbps)
Lidar	Lidar	VCC1	6	1	1500	0 µs	100	No	121.8
Camera	Camera1	VCC1	6	2	490	15 µs	100	No	41
Millimeter-wave radar	Radar1	VCC1	6	6	42	20 µs	1000	Yes	0.5
Camera	Camera2	VCC1	6	7	490	22 µs	100	Yes	41
Millimeter-wave radar	Radar2	VCC1	6	12	42	25 µs	1000	Yes	0.5
Camera	Camera3	VCC1	6	13	490	27 µs	100	Yes	41
Camera	Camera4	VCC1	6	14	490	31 µs	100	Yes	41
Brake	VCC1	Brake_Act1	7	3	42	0 µs	100	No	5.1
Brake	VCC1	Brake_Act2	7	8	42	2 µs	100	Yes	5.1
Brake	VCC1	Brake_Act3	7	15	42	4 µs	100	Yes	5.1
Audio	VCC3	Audio1	4	4	234	0 µs	100–200	No	10.2
Audio	VCC3	Audio2	4	9	234	5 µs	100–200	No	10.2
Audio	VCC3	Audio3	4	16	234	10 µs	100–200	No	10.2
Video	Video	VCC3	4	10	1500	0 µs	100–200	No	60.9
Window	VCC2	Win_Act1	3	5	42	50 µs	100–200	No	2.6–5.1
Window	VCC2	Win_Act2	3	11	42	55 µs	100–200	No	2.6–5.1
Window	VCC2	Win_Act3	3	17	42	60 µs	100–200	No	2.6–5.1

**Table 7:** End-to-end delay of each traffic.

Traffic	Priority	Mean ^1^ (μs)	Min ^1^ (μs)	Max ^1^ (μs)	Jitter ^1^ (μs)	Max ^2^ (μs)	Jitter ^2^ (μs)	Max ^3^ (μs)	Jitter ^3^ (μs)	Differ ^4^ (μs)
Audio1	4	16.78	16.75	18.62	1.87	84.93	68.18	86.01	69.26	67.39
Audio2	4	16.95	16.75	21.33	4.58	85.86	69.11	85.86	69.11	64.53
Audio3	4	11.92	11.89	13.96	2.07	79.87	67.99	79.87	67.99	65.91
Brake_Act1	7	7.30	7.28	9.45	2.17	7.28	0.00	7.28	0.00	−2.17
Brake_Act2	7	10.62	10.60	12.78	2.17	10.60	0.00	10.60	0.00	−2.18
Brake_Act3	7	14.37	13.93	26.25	12.32	13.93	0.00	10.60	0.00	−15.65
Lidar	6	59.19	57.26	61.49	4.23	59.95	0.67	59.28	0.00	−2.21
Camera1	6	48.60	46.52	50.77	4.26	49.20	0.67	48.53	0.00	−2.24
Radar1	6	11.03	10.60	22.93	12.32	10.60	0.00	10.60	0.00	−12.33
Camera2	6	26.31	24.94	43.77	18.83	24.94	0.00	24.94	0.00	−18.83
Radar2	6	22.12	17.26	22.69	5.43	22.61	0.00	13.93	0.00	−8.76
Camera3	6	41.05	40.79	43.03	2.24	41.46	0.67	40.79	0.00	−2.24
Camera4	6	41.31	41.04	43.28	2.24	41.72	0.67	41.04	0.00	−2.24
Video	4	57.36	57.26	61.47	4.21	125.34	68.08	125.34	68.08	63.87
Win_Act1	3	10.97	10.60	16.23	5.62	82.35	71.74	83.37	72.76	67.14
Win_Act2	3	7.31	7.28	9.39	2.12	76.15	68.87	76.15	68.87	66.76
Win_Act3	3	11.24	10.60	23.56	12.96	89.12	78.52	89.12	78.52	65.56

^1^ End-to-end delay without scheduling and without redundancy. ^2^ End-to-end delay with scheduling and without redundancy. ^3^ End-to-end delay with scheduling and with redundancy. ^4^ Difference between Max ^3^ and Max ^1^.

**Table 8:** Time delay requirements of each traffic class.

Traffic Class	Priority	Periodicity	Transmission Period	End-to-End Delay	Tolerance to Loss	Criticality
TC8	Highest	Periodic/Sporadic	≤20 ms	≤100 μs/5 hops	No	High
TC7		Periodic	≤10 ms	≤100 μs/5 hops	No	High
TC6	reserved	/	/	/	/	/
TC5		Periodic	50 ms–1 s	/	Yes	High
TC4		Sporadic	/	≤10 ms	Yes	Medium
TC3		Periodic/Sporadic	≤200 ms	≤50 ms	Yes	Medium
TC2		Periodic	Depends on sensors	≤300 ms	Yes	Medium
TC1	Lowest	Sporadic	/	/	Yes	Low

## Data Availability

Not applicable.
